# Determinants of Mortality Among Older Adults by Area-Level Material and Social Deprivation

**DOI:** 10.1093/geroni/igaa057.892

**Published:** 2020-12-16

**Authors:** Helen-Maria Vasiliadis, Samantha Gontijo Guerra, Isabelle Pitrou

**Affiliations:** 1 University of Sherbrooke, Sherbrooke, Quebec, Canada; 2 University of Sherbrooke, Longueuil, Quebec, Canada

## Abstract

Although social inequalities are increasing worldwide, few studies have examined their consequences on mortality among older adults. The aim of this research was to examine determinants of mortality among older adults at the individual, health system and area level. Data come from the ESA-Services study conducted in 2011-2013 in Quebec including 1,765 adults aged ≥65 years. Mortality 3 years after the survey interview was obtained from vital statistics data. Material and social deprivation of area of residence was determined using the Pampalon index categorized into quintiles as follows: least deprived (1st quintile), middle quintiles (2nd, 3rd, 4th quintiles), most deprived (5th quintile). Other variables included clinical, psycho-behavioural, socio-economic and demographic factors. Cox regressions were used to examine the determinants of mortality while stratifying by level of area deprivation. Compared to most deprived areas, mortality was higher for those living in middle quintiles of deprivation. In the overall analyses, age, chronic conditions, social support and continuity of care were associated with mortality. When examining mortality by area of deprivation, results showed higher mortality ratios with cognitive impairment in middle and least deprived areas. In least deprived areas, female sex, the presence of bipolar disorder and dementia were associated with mortality. The strong associations between mortality, cognition, dementia, bipolar disorder and female sex in least deprived areas might be partially explained by a closer follow-up and earlier detection of this population. Continuity of care was also associated with significantly lower mortality ratios for those living in middle and most deprived areas.

